# Cost effectiveness of strategies for cervical cancer prevention in India

**DOI:** 10.1371/journal.pone.0238291

**Published:** 2020-09-01

**Authors:** Akashdeep Singh Chauhan, Shankar Prinja, Radhika Srinivasan, Bhavana Rai, JS Malliga, Gaurav Jyani, Nidhi Gupta, Sushmita Ghoshal

**Affiliations:** 1 Department of Community Medicine and School of Public Health, Post Graduate Institute of Medical Education and Research, Chandigarh, India; 2 Department of Cytology and Gynaecological Pathology, Post Graduate Institute of Medical Education and Research, Chandigarh, India; 3 Department of Radiation Oncology, Post Graduate Institute of Medical Education and Research, Chandigarh, India; 4 Department of Preventive Oncology, Cancer Institute (WIA), Adyar, Chennai, India; 5 Department of Radiation Oncology, Government Medical College and Hospital, Chandigarh, India; Harvard University T H Chan School of Public Health, UNITED STATES

## Abstract

The establishment of link between high-risk human papillomavirus (HPV) infection and occurrence of cervical cancer has resulted in development of various HPV related control strategies for the prevention of cervical cancer. The objective of the present study was to assess the cost effectiveness of various screening strategies for cervical cancer and human papilloma virus (HPV) vaccination in India. A Markov model based on societal perspective was designed to estimate the lifetime costs and consequences of screening (with either visual inspect with acetic acid (VIA), Papanicolaou test or HPV DNA test at various time intervals) in a hypothetical cohort of 30–65 years age women or vaccination among adolescent girls. Diagnostic accuracy of the screening strategies, efficacy of HPV vaccination and data on transition probabilities was based on the results of the existing meta-analyses. Primary data was collected for assessing per person cost of screening, cost of treating cervical cancer and quality of life. We found that introduction of different screening strategies leads to reduction in lifetime occurrence of cervical cancer cases caused by HPV 16/18 from 20% to 61%, and cervical cancer deaths from 28% to 70%, as compared to no screening. Among various screening strategies, screening with both VIA 5 yearly and VIA 10 yearly came out to be cost effective at 1-time per capita GDP, with VIA every 5 years providing greater health benefits as compared to VIA 10 years. Hence, screening with VIA 5 years at an incremental cost of US$ 829 (INR 54,881) per QALY gained is the recommended strategy for India. Further, with regards to HPV vaccination, it leads to 60% reduction in cancer cases and mortality caused by HPV 16/18 as compared to no vaccination. Moreover, when this vaccinated cohort of adolescent girls is also screened later in their life (with VIA every 10 years and VIA 5 years), it leads to 69%-76% reduction in cancer cases and 71%-81% reduction in cancer deaths. As compared to no vaccination and no screening, both HPV vaccination alone and vaccination plus screening (with VIA every 5 yearly and VIA 10 yearly) appears to be cost effective with ICERs in the range of US$ 86 (INR 5,693) to US$ 476 (INR 31,511) per QALY gained. In the long run, when the cohort of adolescent girls, who were immunized for HPV, reach the age of 30 years, the screening frequency using VIA should be determined based on the coverage of HPV vaccination in that cohort.

## Introduction

Cancer of the uterine cervix is the second most common cancer among women in the developing countries.[1] The establishment of a strong link between high-risk persistent human papillomavirus (HPV) infections and the occurrence of cervical cancer has resulted in development of HPV related control strategies for the prevention of cervical cancer. [2–4] These include interventions ranging from vaccination against HPV for adolescent girls to various screening approaches in the form of visual inspection with acetic acid (VIA) or with lugol’s iodine (VIA/VILI), Papanicolaou test (Pap test) and HPV DNA testing for women later during their reproductive life. [4] Various developed countries have institutionalized Pap cytology test or HPV DNA as primary method of screening, which has in turn led to decline in the annual burden of cervical cancer by 50–70%. [5, 6]. From a macroeconomic point of view, global investment in prevention strategies for cervical cancer could save up to an economic value of US$ 1 trillion, both due to gain in disease free life years as well as with reduction in treatment expenditure. [7, 8]

While the techniques like HPV DNA and cytology based Pap smear has been reported to show high sensitivity and specificity respectively, these are also costly and resource intensive. [4] On the contrary, techniques like VIA/VILI have moderate sensitivity and specificity, but are also less expensive. Various studies have shown its usefulness as affordable and effective methods in the Indian context. [4, 9, 10] The Government of India, under National Program for Prevention and Control of Cancer, Diabetes, Cardiovascular Diseases and Stroke (NPCDCS) has recently initiated a population based screening for cervical cancer (in 100 districts on a pilot basis), using VIA every 5 years for women aged between 30–65 years. [11]

As India is on the path towards universalizing the national level screening program, the present study was designed to assess the cost-effectiveness of three strategies for screening cervical cancer among women in the age group of 30–65 years—VIA, Pap smear and HPV DNA. The costs and benefits of each of the 3 type of tests were evaluated if applied at population level at a frequency of every 3 years, 5 years and 10 years respectively. In addition, we evaluated the cost effectiveness of 2 alternative scenarios—introducing HPV vaccination alone, and a combination of screening and vaccination.

## Methodology

### Model overview

We undertook a model-based cost-utility analysis for estimating the lifetime costs and consequences in a hypothetical cohort of 30 year old women undergoing screening using a societal perspective. Specifically, for the scenario of HPV vaccination, a cohort of 11 year pre-adolescent girls was used in the decision model. The cycle length of the model was taken as 1 year. Future costs and consequences were discounted at the rate of 3% from 30 years onwards in the case of screening and 11 years onwards in the case of vaccination. The outcomes were measured in terms of reduction in cancer incidence, mortality, life-years (LYs) and quality adjusted life years (QALYs).

**Fig 1:**
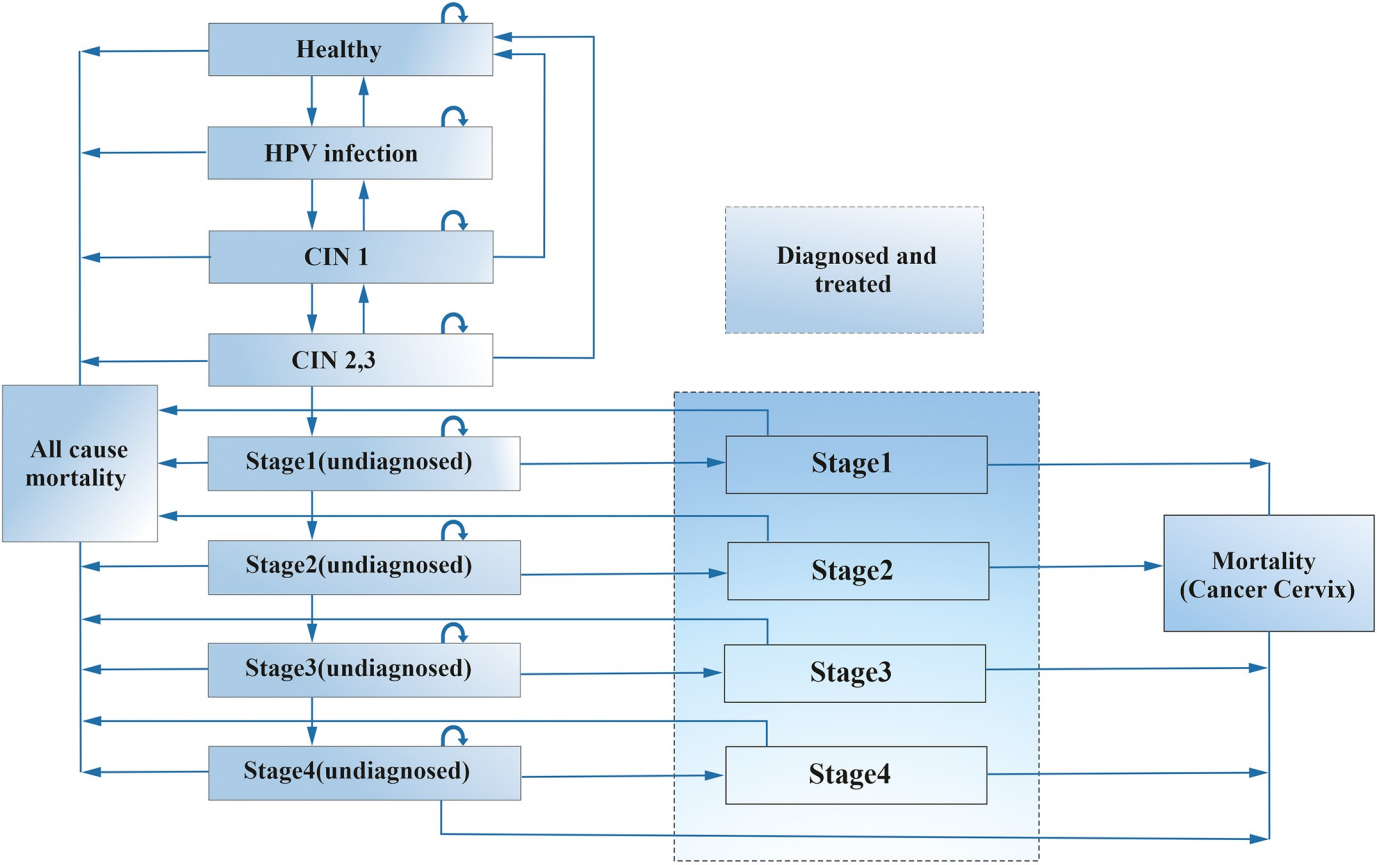
Markov model.

Based on the previously published and validated models for cervical cancer, we developed a markov model on MS Excel spread sheet, considering the natural history of HPV infection and cervical cancer (Fig 1). [12–15] The markov health states are denoted in rectangle boxes and the arrows from one box to another indicates the annual probability of transition or movement from one health state to another. The arrow from a rectangular back into itself shows the likelihood of remaining in the same health state. As per the model, the women with no infection (healthy state) can get an HPV infection or remain in the same state in the next cycle. Further, the women infected with HPV can develop precancerous state i.e., cervical intra-epithelial neoplasia 1 (CIN1; low-grade squamous intraepithelial lesion) and CIN2/CIN3 (high-grade squamous intraepithelial lesion), who can in turn move back to the previous healthy state or can remain in the same precancerous state during the next cycle. The persistent HPV infection can transform into invasive cancer. Once a woman develops invasive cancer, she cannot return to the previous or a healthy state, but can only progress to next advanced cancerous stage in the subsequent cycle of the model or remain in the same stage. [12, 16–19] The progression to more advances invasive stages is dependent on the probability to get diagnosed and treated. Finally, the patient can die (from each of the health state) from causes other than cervical cancer as per age-specific all-cause mortality rates [20] or due to cervical cancer (in invasive cancer state) as per mortality rates of an untreated and treated cervical cancer. [12, 16] It was assumed that patients in undiagnosed cervical carcinoma can die due to cancer, only after progressing through all the stages of the cancer (as per natural history of the cervical cancer) and within the first year of moving into the stage 4.

The present model did not consider all infections due to various HPV types separately, as the parameters used were specific to high-risk HPV types i.e., 16 and 18. Together, these two HPV types account for around 85% of the cervical cancer cases in India. [21] Considering that utility of screening is through the early detection of precancerous lesions or those in those in the early stages of cancer, it was assumed that women in precancerous stage could be detected only through screening (based on the sensitivity of the screening strategy) and those in the invasive cancer stage could be detected both either through the screening or by the onset of symptoms. [10, 22] Further, the utility of vaccination was based on its efficacy in providing immunity to the HPV infection. The invasive cancer was assumed to be treated according to the India’s National Cancer Grid Guidelines for the treatment of invasive cervical cancer. [23, 24] Similarly, the precancerous lesions were assumed to be treated as per standard guidelines i.e., with cryotherapy, loop electrosurgical excision procedure (LEEP) or surgery depending upon the spread of the lesion (S1 File of S1 Table). [9, 22, 25] Women treated for precancerous lesions were considered treated for HPV infection and were assumed to return to the healthy state, but were still at risk for future disease based on the age specific incidence of HPV infection.

We compared introduction of 3 screening strategies i.e., VIA, Pap smear and HPV DNA test, each at 3 different screening interval of every 3 years, 5 years and 10 years among women in the age group of 30–65 years, as compared to no screening. Further, the cost effectiveness of 2 additional scenarios of HPV vaccination alone, and combination of vaccination and screening (HPV vaccination at 11 years followed by screening women who were eligible for vaccination when they were adolescents with the most cost effective screening strategy later in the life) were assessed. The age group of 30 to 65 years for the purpose of screening was as per India’s NPCDCS guidelines. [11] Following the same guidelines, screening was assumed to be undertaken at the level of sub-centers by the auxillary nurse midwives, supported and supervised by the concerned lady health visitor/staff nurse. [11] It was also assumed (as per guidelines) that screening would be done on the fixed days preceded by the awareness campaigns to ensure high level of participation. [11] While the results of screening with VIA were immediately available, the results of screening with Pap smear and HPV DNA test were assumed to be available at 2 weeks following screening. Those screened positive with either of the screening strategy are offered colposcopy/biopsy at the level of community health center (CHC) or district hospital (DH). Finally, for the treatment of the precancerous and cancerous lesions, patients were assumed to be referred to the DH and tertiary care hospital respectively. Based on the previous feasibility studies conducted across India, coverage of screening for each of the screening strategy and HPV vaccination was assumed as 80% and 70% respectively. [9, 10, 22] Further, it was assumed that there would be a loss of 10% each from those screened positive to undergoing colposcopy, and subsequent treatment respectively.

As per care seeking behavior in the scenario of no screening, it was assumed that women diagnosed of invasive cancer would avail treatment from a mix of public and private health care facilities based on utilization pattern (40% and 60% in public and private facilities respectively) reported from National Sample Survey (NSS) 2104–15. [26] However, in the scenario of organized population based screening, women diagnosed of invasive cancer were systematically referred and treated in a public sector tertiary care hospital. As per HPV vaccination experience in India, [27] the cohort of pre-adolescent girls were assumed to be administered with 2 doses of bivalent vaccine (covering HPV-16 and HPV-18 strain) along with routine immunization at health facilities.

### Model parameters

Using the reported annual incidence rate of 0.8% for the HPV infection (HPV 16 and 18) among 20–25 year old immunized women with 2 doses of HPV vaccine [28] and vaccine efficacy of 93%, [29] we computed the incidence rate of HPV infection as 11.6% among unvaccinated cohort of the same age group. Further, using the differential of prevalence of HPV infection among other age groups relative to 20–25 year old, we estimated the age specific incidence of HPV infection till 50 years of age (Table 1). Beyond 50 years of age, prevalence of HPV infection gets increased by more than 2 fold. [21] We used incidence rate of 0.005 among those beyond 50 years of age as derived from a previously published mathematical model [16] and calibrated it to Indian specific incidence, based on the percentage difference in the incidence in the preceding age groups as derived in the present model to that of the reported incidence in the mathematical model (S1 File of S2 Table).

**Table 1:** Selected model inputs: Baseline values.

Parameters	Categories	Base value	Standard error	Source	Distribution
**Prevalence among 30 year old women in India**	HPV infection	0.07	0.00714	[21, 30]	Beta
	CIN 1	0.0168	0.00408		
	CIN 2,3	0.00414	0.00092		
	Invasive cervical cancer	0.000085	0.000010		
**Incidence of HPV infection among Indian women (age in years)**	30–34 years	0.06	0.00612	[28, 29]	Beta
	35–39 years	0.047	0.00480		
	40–44 years	0.047	0.00480		
	44–49 years	0.046	0.00469		
	50 years and above	0.0125	0.00128		
**Annual progression probabilities**	HPV infection to CIN 1	0.078	0.01592	[19, 31]	Beta
	CIN 1 to CIN 2/3^@^	0.046	0.01448		
	CIN 2/3 to invasive cancer stage 1	0.072	0.01469		
	Stage 1 to stage 2	0.438	0.08939	[16]	
	Stage 2 to stage 3	0.536	0.10939		
	Stage 3 to stage 4	0.684	0.13959		
**Annual regression probabilities**	CIN 2/3 to CIN 1	0.055	0.01122	[16, 19, 31]	Beta
	CIN 1 to HPV infection	0.082	0.01673		
	CIN 2/3 to normal (without HPV infection)	0.085	0.01735		
	CIN 1 to normal (without HPV infection)	0.142	0.02898		
**Proportion showing symptoms**	Stage 1	0.127	0.01297	[16]	Beta
	Stage 2	0.191	0.01946		
	Stage 3	0.578	0.05901		
	Stage 4	0.867	0.08851		
**Annual mortality rates**	Stage 1	0.025	0.00255	[32]	Beta
	Stage 2	0.078	0.00796		
	Stage 3	0.141	0.01439		
	Stage 4	0.444	0.04531		
**Health state utility values**	Stage 1	0.698	0.04210	a	Beta
	Stage 2	0.632	0.02257		
	Stage 3	0.637	0.04269		
	Stage 4	0.591	0.09074		
**Sensitivity of the screening/diagnostic test**	Visual inspection with acetic acid	0.676	0.069	[33, 34]	Normal
	Pap smear	0.621	0.063		
	HPV DNA	0.778	0.079		
	Colposcopy	0.95	0.0242		
**Specificity of the screening/diagnostic test**	Visual inspection with acetic acid	0.843	0.021		
	Pap smear	0.935	0.0238		
	HPV DNA	0.915	0.0233		
	Colposcopy	0.42	0.0107		
**Cost of screening (US$)**	Per women screened with visual inspection with acetic acid	5.2	1.3	a	Gamma
	Per women screened with Pap smear	9.8	2.5		
	Per women screened with HPV DNA	14.8	3.8		
**Cost of Vaccination (US$)**	Cost of two doses of HPV vaccine per girl	8.8	2.2	[35]	Gamma
	Service delivery cost of HPV vaccine per girl	5	1.3		
**Cost of treatment of precancerous lesions (US$)**	Per patient cost for colposcopy	16.6	4.2	[36]	Gamma
	Per patient cost for biopsy	31.2	8		
	Per patient treated with cryotherapy	60.4	15		
	Per patient treated with loop electrosurgical excision procedure	90.3	23		
**Health system cost of treating invasive cancer (US$)**	Outpatient consultation and diagnostics	127.8	33	a	Gamma
	Surgery	295	75		
	Radiotherapy (3-dimensional radiotherapy)	625	159		
	Brachytherapy	406	104		
**OOP expenditure in public hospitals for the treatment of invasive cancer (US$)**	Outpatient consultation and diagnostics	161	12	a	Gamma
	Surgery	354	90.3		
	Radiotherapy (3-dimensional radiotherapy)	194	8.6		
	Brachytherapy	84.3	5		
	Chemotherapy	61	4.8		
	Before visiting tertiary care facility	241	241		
**OOP expenditure in private hospital for treating invasive cancer (US$)**		1,181	301	[26]	Gamma

*HPV: Human papillomavirus; CIN: cervical intra-epithelial neoplasia; a: primary data; OOP: Out of pocket expenditure; US$: Unites States Dollar

^@^Parameter value was calibrated based on lifetime risk of developing cervical cancer

The prevalence of HPV infection, precancerous lesions and invasive cancer among 30 year old women was based on the data from Indian cancer registries and other primary studies.[21, 30] The natural history parameters including annual probabilities of progression or regression in an unscreened population were derived from the literature as shown in Table 1. Specifically, the probability of progression from HPV infection to precancerous states or invasive cancer and regression to previous or normal stage was based on the pooled estimates of 2 meta-analyses studies. [19, 31] Further, the data on probability of progression from an undiagnosed stage of cancer to the next advanced stage was based on a mathematical model on the natural history of HPV infection and cervical cancer. [16] Similarly, the proportion of patients getting diagnosed in any stage of the cancer was estimated based on the probability of occurrence of cancer specific symptoms in the respective state, as reported from the same mathematical model. [16] As the likelihood of showing symptoms and finally getting diagnosed is dependent on the extent of unmet need, and other factors related to availability of health care, we scaled down the value of those showing cancer specific symptoms with the prevalence of unmet need (3.62%) and those availing cancer treatment from the informal sector (11.64%) as reported in the Indian NSS (2014–15) survey. [26] Lastly, the stage-specific survival rates were determined from an Indian randomized control trial (RCT) in which patients were followed up to 14 years [32]. The probability of age-specific all-cause mortality was obtained from the Census of India Sample Registration System life tables for the female population. [20]

Sensitivity and specificity of each of the screening strategy and colposcopy and efficacy of HPV vaccine was based on the published meta-analysis studies (Table 1). [29, 33, 34] While the sensitivity of diagnosing stage 1 of the cancer was assumed to be same as that of the precancerous states, the sensitivity was assumed to be 100% for diagnosing women in stage 2 to stage 4 of invasive cancer. It was also assumed that the biopsy always resulted in the diagnosis of true health state.

### Cost data

Primary data was collected using bottom up micro-costing methods [37] from a population based screening program conducted in the Villupuram district of Tamil Nadu in 2016–17, for estimating the cost of screening. Methodological details of cost data collection is shown in S2 File. Unit cost of each of these 3 screening strategies inclusive of sample collection, laboratory processing and support activities (IEC activities, administration, documentation, travel, etc.) are shown in Table 1. Per girl vaccinated cost was taken as US$ 13.9 (INR 918) as estimated in an earlier recent study. It comprised of both the vaccine cost (US$ 8.8; INR 586) as well as the service delivery cost (US$ 5; INR 332). [35] The service delivery cost consisted of opportunity cost of human resource time and expenditure on capital items, consumables, vaccine storage and its transport.

The cost of treatment for cervical cancer was based on the primary data collected from a large public sector tertiary care hospital in North India for the year 2016–17. [38] Following the standard bottom up and economic costing methods, health system cost of surgical hysterectomy, radiotherapy, chemotherapy and brachytherapy for the treatment of cervical cancer was estimated (Table 1). In addition, OOP expenditure incurred by the patients on various therapeutic interventions was elicited by interviewing a sample of 237 patients. Indirect expenditure due to wage loss was not included in our analysis. The reimbursement rates of Central Government Health Insurance scheme (CGHS) were used for assessing the cost of colposcopy, biopsy, cryotherapy, LEEP and palliative care. [36] The detailed methodological note on assessing cost of cervical cancer treatment has been provided as S3 File. All the costs are reported both in United States Dollar (US$) and Indian National Rupees (INR) as per conversion rate for the year 2016–17 (1 US$ = 66.2 INR).

### Health state utility values

A total of 223 cervical cancer patients were recruited from the radiotherapy department of a tertiary care hospital in north India for assessing the health related quality of life (HRQoL) using standard EQ-5D-5L tool. The patients between 18–70 years age, who had undergone treatment for histologically proven cervical cancer, after being diagnosed in any of the stage I-IVb (FIGO classification) were included. Based on the consultation with the oncologists, it was assumed that HRQoL tends to get stabilized after 4–5 months following treatment. Thus, those patients who had completed at least 4 months post treatment for cervical cancer were considered eligible and were interviewed at the time of their follow-up visit in the outpatient clinic of radiotherapy department.

### Sensitivity analysis

To test the uncertainty in the parameter values, we undertook multivariate probabilistic sensitivity analysis (PSA) to account for joint parameter uncertainty. [39] Under PSA, each of the parameters was assigned specific distribution based on its nature. Specifically, gamma distribution was assigned to cost parameters and beta distribution was used for HRQoL estimates and other parameters reported as rates, proportion and probabilities. All the health system cost estimates were varied from half to double of the base value. Standard error for OOP expenditure and HRQoL was based on the results of the primary data. Epidemiological parameters in the form of prevalence, incidence and mortality were varied by 20% on either side of base case value. Similarly, annual probabilities of progression and regression were varied by 40% on either side of the base value. Given the extent of variation seen in the sensitivity of screening tests among studies included in the meta-analysis, we varied it by 20% on either of the base value. Further, since the estimate of specificity was already more than 90% (for HPV and Pap smear) and in view of small variation in its estimates among various studies, it was varied by 5% of the base value. Finally, the median value of incremental cost effectiveness ratio (ICER) along with 2.5^th^ and 97.5^th^ percentile was computed using 999 Monte Carlo simulations.

To assess the comparative cost effectiveness between the various screening strategies, concept of dominance and extended dominance was used. [40–42]. We also undertook specific threshold analysis to assess the minimum coverage of treatment for screen positives, as well as lifetime risk of cervix cancer/incidence of HPV infection necessary to maintain cost-effectiveness of screening. A subgroup analysis was undertaken to determine the impact of screening among poor (bottom 1/3^rd^ of the income group) and non-poor population (upper 2/3^rd^ of the income group), based on odds of occurrence of the incidence of HPV infection among the respective income groups. [43]

### Ethical approval

Ethical approval was obtained from the Institute Ethics Committee of the Post Graduate Institute of Medical Education and Research, Chandigarh, India with reference number: IEC-12/2017-786. All the respondents during primary data collection were interviewed after obtaining written informed consent.

## Results

### Screening

#### Health outcomes

As per model, a total of 2,090 cervical cancer cases and 1,650 cancer deaths occurred due to HPV 16/18 during the lifetime among a cohort of 100,000 women, in case no screening and no vaccination is undertaken. This implies a 2.09% lifetime risk of developing cervical cancer among Indian women (Table 2). Decline in the number of cancer cases was observed with introduction of screening, which varied from 20% (n = 414) to 61% (n = 1280) respectively with different strategies. While lowest reduction in cancer cases was observed for Pap smear every 10 years, the highest benefits in terms of reduction in cancer cases was found to be with HPV DNA based screening done every 3 years. Similarly, percentage decrease in cancer deaths with use of screening varied from 28% (n = 456) to 70% (n = 1163), which was lowest for Pap smear every 10 years and highest in case of HPV DNA based screening done every 3 years. This reduction in cancer cases and associated mortality translated into gain of 3,517 to 8,107 life years and 3,887 to 9,437 QALYs among various strategies.

**Table 2:** Lifetime health consequences in a cohort of 100,000 women (aged 30 years) among various screening scenarios.

Screening strategy		Cancer cases*	Deaths*	Cancer cases averted#	Deaths averted#	Life years gained*	QALY gained*
**No organized screening**		2090 (1104–3576)	1650 (873–2824)	-	-	-	-
**Visual inspection with acetic acid**	**3 Years**	896 (462–1639)	530 (278–986)	1167 (56)	1099 (67)	7614 (4237–13,446)	8789 (4825–15,462)
	**5 Years**	1210 (622–2140)	771 (407–1400)	851 (41)	860 (52)	5938 (3245–10,683)	6770 (3670–12,216)
	**10 Years**	1590 (820–2740)	1123 (586–1953)	474 (23)	512 (31)	3815 (2116–6857)	4255 (2321–7626)
**PAP smear**	**3 Years**	998 (481–1842)	598 (291–1115)	1066 (51)	1031 (62)	7208 (3782–13,034)	8262 (4294–15,081)
	**5 Years**	1312 (652–2372)	843 (415–1530)	757 (36)	790 (48)	5550 (2896–10,103)	6233 (3203–11,541)
	**10 Years**	1664 (859–2934)	1182 (606–2094)	414 (20)	456 (28)	3517 (1880–6385)	3887 (2021–7189)
**HPV DNA test**	**3 Years**	772 (392–1443)	452 (233–864)	1280 (61)	1163 (70)	8107 (4488–14,243)	9437 (5212–16,697)
	**5 Years**	1084 (523–1970)	682	966 (46)	932 (56)	6470 (3550–11,607)	7406 (4048–13,204)
	**10 Years**	1516	1054	559 (27)	579 (35)	4257 (2418–7697)	4763 (2711–8663)

* Values in parenthesis represent 2.5^th^ and 97.5^th^ percentile,

^#^ Figure in parenthesis indicate percentage decrease in cancer cases and deaths due to HPV 16/18 with various screening strategies as compared to the scenario of no screening; Pap: Papanicolaou test; QALY: Quality adjusted life year

#### Cost

The lifetime cost incurred by the cohort of 100,000 women in the scenario of no screening was US$ 2.45 million (INR 163 million), which was mainly (85%) on account of treatment expenditure for invasive cancer (US$ 2.08 million; INR 138 million) (Table 3). Similarly, the overall cost incurred in case of various screening scenarios ranged from US$ 4.82 million (INR 319 million) to US$ 16.64 million (INR 1101 million). The implementation of VIA every 10 years was cheapest strategy, while use of HPV DNA every 3 years was the costliest strategy. Among the various screening scenarios, the cost of implementation of screening program constituted 69% (US$ 3.34 million; INR 221 million) to 95% (US$ 15.74 million; INR 1042 million) of this overall cost. The cost of providing treatment to cancer cases ranged from 5% (US$ 0.80 million; INR 53 million) to 31% (US$ 1.48 million; INR 98 million) of the total cost of different screening scenarios. The decline in treatment cost due to reduction in number of cases among various screening scenarios led to savings in terms of lifetime reduction in per capita (women) OOP expenditure ranging from US$ 7.97 (INR 527) to US$ 9.96 (INR 659) (S1 File of S3 Table). The distribution of cost in terms of health system cost and OOP expenditure among various screening scenarios has been mentioned in the S4 Table of S1 File.

**Table 3:** Total cost incurred with implementation of various screening strategies.

Screening strategy		Screening cost in million		Treatment expenditure in million		Total cost in million*	
		INR	US$	INR	US$	INR	US$
**No organized screening**		24 (16–34)	0.38 (0.20–0.50)	138 (72–248)	2.08 (1.10–3.7)	163 (96–275)	2.45 (1.45–4.16)
**Visual inspection with acetic acid**	**3 Years**	726 (521–972)	10.97 (7.90–14.70)	61 (32–109)	0.93 (0.50–1.65)	789 (576–1046)	11.92 (8.70–15.80)
	**5 Years**	380 (276–503)	5.74 (4.16–7.60)	80 (41–136)	1.20 (0.6–2.06)	462 (348–599)	6.98 (5.26–9.04)
	**10 Years**	221 (162–291)	3.34 (2.4–4.4)	98 (48–176)	1.48 (0.75–2.5)	319 (241–417)	4.82 (3.64–6.3)
**PAP smear**	**3 Years**	787 (556–1103)	11.88 (8.40–16.67)	67 (34–127)	1.02 (0.51–1.92)	855 (621–1172)	12.91 (9.37–17.71)
	**5 Years**	418 (289–595)	6.31 (3.36–9.0)	85 (43–155)	1.28 (0.65–2.34)	508 (363–692)	7.67 (5.48–10.45)
	**10 Years**	241 (166–340)	3.64 (2.50–5.13)	102 (51–186)	1.54 (0.77–2.81)	345 (246–471)	5.21 (3.72–7.12)
**HPV DNA test**	**3 Years**	1042 (763–1436)	15.74 (11.53–21.70)	53 (28–97)	0.80 (0.42–1.46)	1101 (813–1849)	16.64 (12.30–22.50)
	**5 Years**	574 (410–803)	8.67 (6.20–12.13)	72 (37–130)	1.09 (0.56–1.96)	647 (479–880)	9.77 (7.24–13.29)
	**10 Years**	326 (238–450)	4.93 (3.59–6.80)	93 (49–170)	1.40 (0.74–2.56)	423 (318–570)	6.39 (4.80–8.60)

*Total cost in a cohort of 100,000population; Pap: Papanicolaou test; Values in parenthesis represent 2.5^th^ and 97.5^th^ percentile; US$: Unites States Dollar; INR: Indian rupees

#### Cost effectiveness

Screening with Pap smear at any frequency was dominated by other screening strategies as shown in Table 4. Further, HPV DNA testing every 5 years was extendedly dominated by screening strategy of VIA every 3 years and VIA 5 yearly. Similarly, HPV DNA testing every 10 years was extendedly dominated by screening strategy of VIA every 5 years and VIA 10 yearly Finally, among the non-dominated strategies, VIA every 5 years was found to be the most cost-effective strategy (below GDP per capita of US$ 1890 in the year 2016–17 for India) with maximum health gains at an incremental cost of US$ 829 (INR 54,881) per QALY gained.

**Table 4:** Cost effectiveness of screening strategies.

Strategy	Cost per women in US$ (INR)	QALY per women	Incremental cost in US$ (INR) per QALY gained	Status
**No screening**	25 (1627)	23.6418		ND
**VIA: 10 years**	48 (3192)	23.6837	564 (37,339)	ND
**VIA: 5 Years**	70 (4621)	23.7097	829 (54,881)	ND
**VIA: 3 Years**	119 (7890)	23.7311	2310 (152,947)	ND
**HPV: 3 Years**	166 (11,013)	23.7334	20,151 (1,334,010)	ND
**Pap: 10 Years**	52 (3450)	23.6786		D
**Pap: 5 years**	77 (5078)	23.7022		D
**Pap: 3 years**	129 (8545)	23.7223		D
**HPV: 10 Years**	64 (4231)	23.6850		ED
**HPV: 5 Years**	98 (6469)	23.7130		ED

*VIA: Visual inspection with acetic acid; Pap: Papanicolaou test; D: Dominated; ND: Non-Dominated; ED: Extended Dominance; ICER: incremental cost effectiveness ratio; QALY: Quality adjusted life years; US$: Unites States Dollar

#### Sensitivity analysis

**Fig 2:**
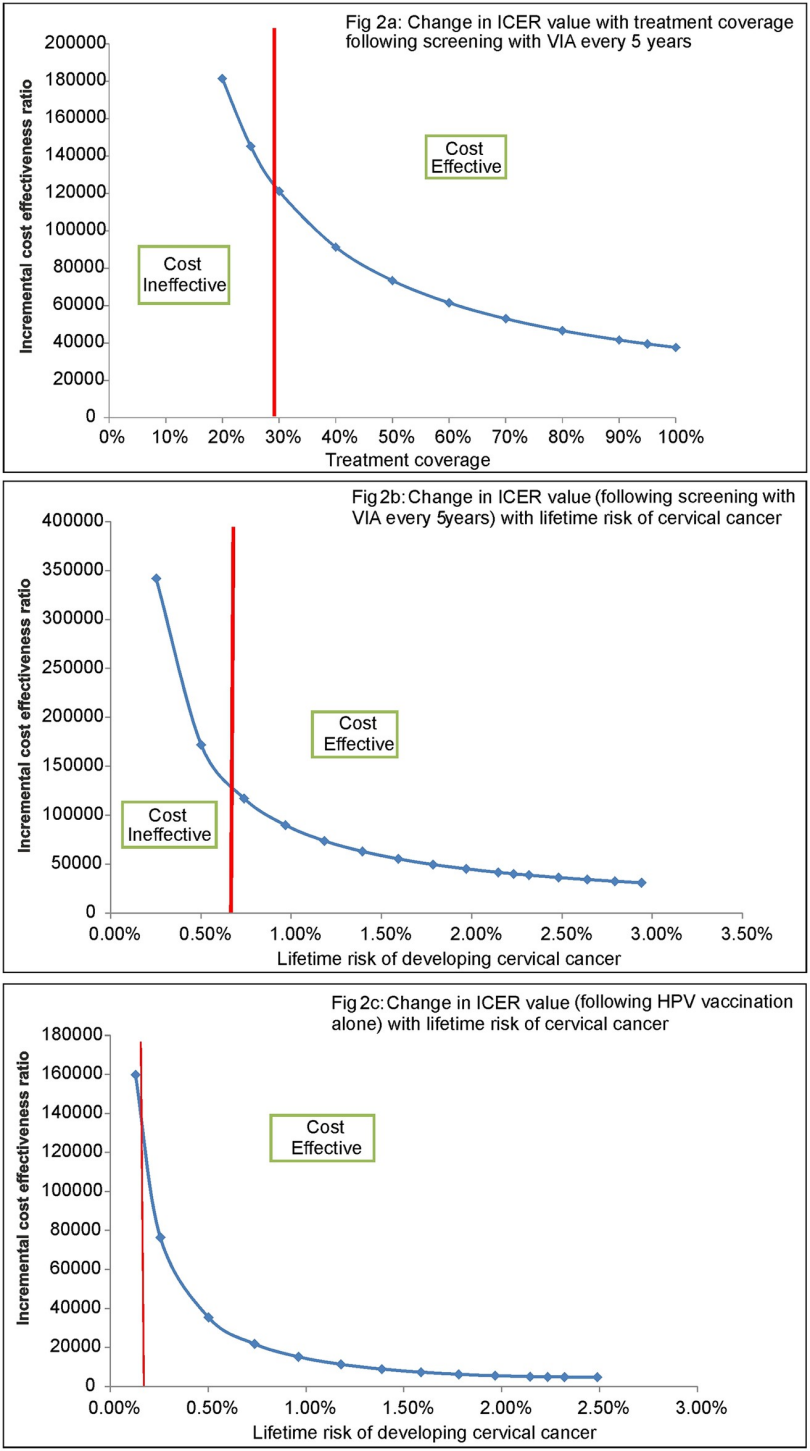
Threshold analysis.

It was seen that, if the treatment coverage (of those screened positive) following screening goes down below 30%, screening strategy of VIA every 5 years ceases to be cost effective (Fig 2a). Similarly, lifetime risk of cervical cancer of at least 0.70% is required for VIA every 5 years to remain cost effective (Fig 2b). Likewise, screening with VIA 5 years ceases to become cost effective, when sensitivity of VIA falls below 17% (S1 File of S1 Fig). Further, it was also seen that there was around 35% greater reduction in cervical cancer cases and subsequent mortality among women belonging to bottom 1/3rd of the income population group as compared to upper 2/3rd of the income group in India, with implementation of screening strategy of VIA every 5 years (S1 File of S2 Fig).

### HPV vaccination

Introduction of HPV vaccination alone led to a 60% decline in cervical cancer cases and related mortality caused by HPV 16/18 in the lifetime of the cohort of 100,000 adolescent girls (aged 11 years), as compared to no vaccination and no screening (Table 5). Moreover, when this cohort of adolescent girls was also screened later in their life with VIA every 5 years, it led to 76% reduction in cancer cases and 81% reduction in cancer deaths. In contrast, screening the same cohort with VIA every 10 years led to 69% reduction in cancer cases and 71% reduction in cancer deaths. This decline in cancer incidence and mortality resulted in a gain of 5,693 and 7,424 QALYs at an additional cost of US$ 0.48 million and US$ 3.52 million with implementation of vaccination alone and in combination with screening.

**Table 5:** Health outcomes and incremental cost effectiveness ratio of introducing HPV vaccination alone and along with VIA every 5 years.

Scenarios	Cancer cases averted*	Deaths averted*	QALYs gained^#^	Incremental cost in US$ million^#^	Incremental cost (US$) per QALY gained#		
					Compared to no vaccination	Compared to vaccination alone	Compared to vaccination plus screening with VIA 10 yearly
**HPV Vaccination only**	1,239 (60)	979 (59.8)	5,693 (3,340–9,741)	0.48 (-0.35 to 1.20)	86 (-44 to 311)	-	-
**HPV vaccination along with screening with VIA 10 yearly**	1,419 (69)	1,167 (71.3)	6,793 (3,941–11,518)	2.25 (1.07 to 3.36)	402 (128 to 856)	1,641 (711–3,462)	-
**HPV vaccination along with screening with VIA 5 yearly**	1,577 (76)	1,331 (81.3)	7,424 (4,266–12,758)	3.52 (2.14 to 5.05)	476 (193 to 979)	1,754 (826–3,823)	1,986 (956–4,417)

*The value indicate comparison as to the scenario of no screening and no vaccination in a cohort of 100,000 population girls aged 11 year; *Values in parenthesis indicate percentage decrease in cancer cases and deaths;

^#^Values in parenthesis represent 2.5^th^ and 97.5^th^ percentile; US$: Unites States Dollar

**Fig 3:**
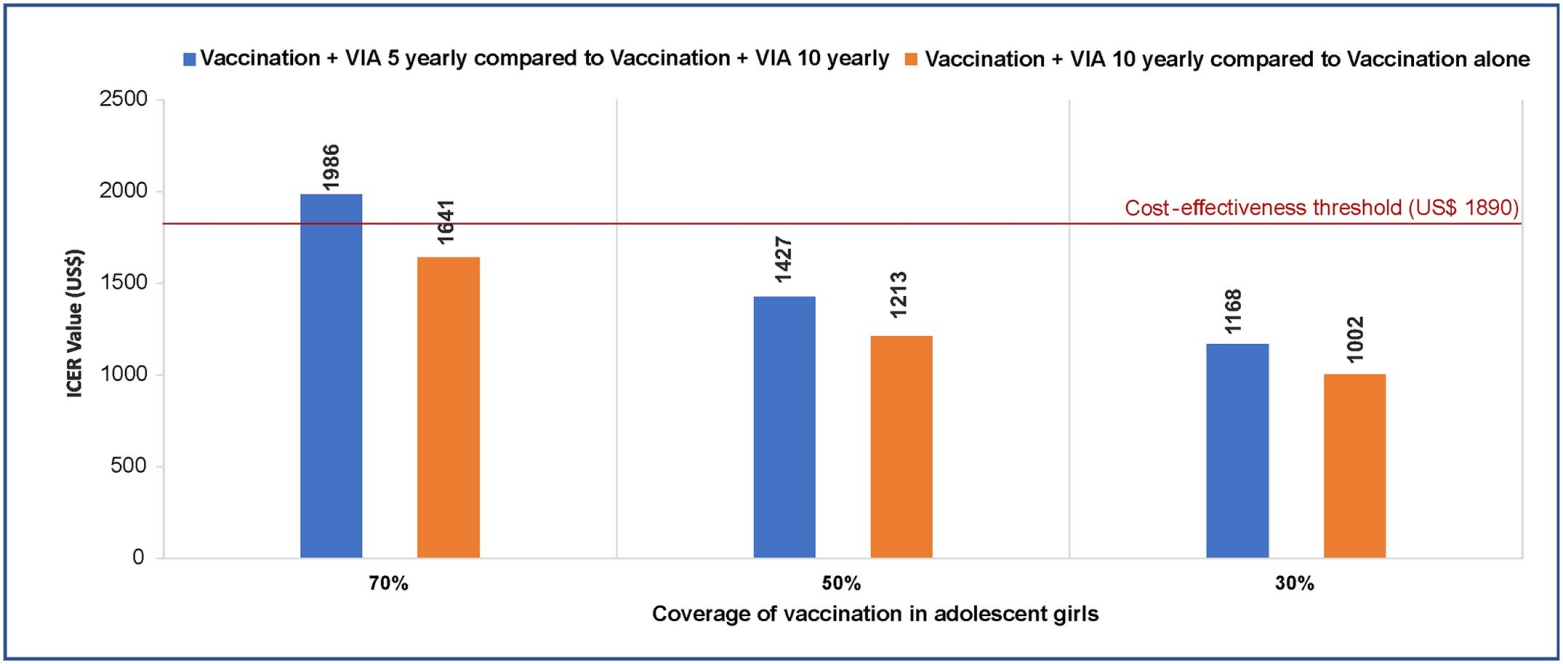
Incremental cost- effectiveness ratios of combination of vaccination and screening at different levels of coverage of HPV vaccination.

The incremental cost per QALY gained with implementing vaccination alone was US$ 86 (INR 5693) as compared to the scenario of no vaccination and no screening. Similarly, when vaccinated cohort is also screened with VIA 5 yearly and VIA 10 yearly, it leads to incremental cost effectiveness ratio (ICER per QALY gained) of US$ 402 (INR 26,212) and US$ 476 (INR 31,511), respectively as compared to no vaccination and no screening (Table 5). Further, as compared to vaccination alone, vaccination plus screening with VIA 5 yearly and VIA 10 yearly results in an incremental cost of US$ 1,754 (826–3,823) and US$ 1,641 (711–3,462) per QALY gained respectively. Analysis of extended dominance reflects that as compared to vaccination plus screening with VIA every 10 years, the strategy of vaccination plus screening with VIA every 5 years results in an incremental cost of US$ 1,986 (956–4,417) per QALY gained, which is more than the cost- effectiveness threshold equals to GDP per capita for India. However, if HPV vaccination coverage is 50% and 30%, respectively, the strategy of vaccination plus screening with VIA every 5 years also becomes cost effective with an incremental cost of US$ 1,427 and US$ 1,168 per QALY gained, as compared to vaccination plus screening with VIA every 10 years (Fig 3).

HPV vaccination alone ceases to be cost-effective, when the lifetime risk of developing cervical cancer falls below 0.15% (Fig 2c). The cost summary of introducing the scenario of HPV vaccination alone and in combination with screening has been presented in S5 Table of S1 File.

## Discussion

Experience from developed countries has shown that prevention measures in the form of screening and vaccination is effective as well as cost-effective in reducing the burden of cervical cancer. [5] But limited availability of infrastructure and trained manpower in developing country like India, poses both financial challenge as well as the issue of health system feasibility in implementing these preventive interventions. The present study was designed to assess the cost effectiveness of available screening strategies and HPV vaccination in the context of India. We conclude that as of now for the NPCDCS program of the Government of India for the screening of women between 30–65 years, VIA at the frequency of 5 years is a cost- effective strategy at an incremental cost of US$ 476 per QALY gained. Introduction of HPV vaccination among adolescent girls was also found to be very cost- effective for prevention of cervical cancer. As compared to no vaccination and no screening, immunizing adolescent girls for HPV along with screening women using VIA appears to be a cost- effective strategy at both 5 yearly and 10 yearly frequencies. In the long run, when the cohort of adolescent girls, who were immunized for HPV, reach the age of 30 years, the screening frequency using VIA should be determined based on the coverage of HPV vaccination in that cohort (Fig 3). States with lower levels of HPV vaccination coverage should continue to use the 5 yearly frequency to screen women later in their life using VIA. Whereas, states with high level of HPV vaccination coverage could consider a lower frequency, i.e., 10 yearly, to screen women for cervical cancer using VIA.

### Model validation

In order to validate the estimates, we compared the outcomes from the present study with the existing epidemiological data and other published evidence. The cost per women screened using different methods estimated by us are very similar in terms of the extent and pattern to a previous study conducted by Legood et al in 2005 (S1 File of S6 Table). [44–46] However, our cost estimates were higher than what was reported by Diaz et al and Goldie et al even after adjustment of latter estimates for inflation since the year of estimation. [47, 48]. One reason for this discrepancy might be due to the non-inclusion of cost pertaining to information, education and communication (IEC) activities in these previous studies. In our study, this cost constituted a large proportion of total cost, ranging from 70% in case of VIA to 24.6% in case of HPV DNA (S2 File). As IEC activities play an instrumental role in success of a screening program, especially when the program is thought to be launched for the first time on a countrywide basis, it is has to be essentially included in the calculation of overall cost.

Based on the data from cancer registries of India, International Agency for Research on Cancer (IARC) has reported a cumulative lifetime risk (%) of developing cervical cancer in India as 2.40%. [21] Our model predicted this risk as 2.09%. Considering that our model was calibrated to predict risk of cervical cancer as a result of high risk HPV variants, which have been reported to constitute 85% of the total burden, the findings on valuation of consequences in our model for no screening scenario are validated. Upon screening with VIA, cytology and HPV DNA every 10 years, we estimated a mean cancer reduction of 26%, 23% and 30% respectively. Using an individual based stochastic model for India, Diaz et al (2008) reported mean cancer reduction as 29%, 21% and 33%, when women were screened thrice per lifetime with VIA, cytology and HPV DNA, respectively. [47] This implies that our estimates on health outcomes are on similar lines as predicted by the previous cost effectiveness model.

Our study concluded that VIA performed at the frequency of every five years yields the best value for money and hence is most cost-effective strategy as compared to both Pap smear and HPV DNA test. A systematic review of economic evaluations on cervical cancer screening conducted across low and middle income countries (LMIC), also concluded that VIA or HPV are the most efficient alternatives for screening and cytology based screening was shown to be the least effective and more costly screening method. [49] Finally, our finding regarding the cost effectiveness of HPV vaccination are in line with a previous analysis done for Punjab state in India. [35] We improved the present analysis as compare to the previous publication by adapting the model structure using long term mortality data from India. Moreover, primary data was collected for estimating cost of treatment and quality of life valuation. With these improvements, our estimates further validate the previous evidence.

### Strengths and limitations

Following the standard guidelines of an economic evaluation, the effectiveness estimates in terms of sensitivity and specificity of the screening strategies was based on the recently published meta-analysis of Indian studies. [33] Similarly, most of the probabilities of progression and regression for the natural history HPV based cancer cervix were based on the meta-analysis of international studies. [19, 31] Another strength of our study was the use of local data on the cost of screening, treatment of cervical cancer and HRQoL valuation. Our cost analysis captures the realistic programmatic guidelines of NPCDCS program. While estimating the cost of cancer treatment, both the health system cost as well as OOP expenditure was estimated following standard methodologies [37, 40, 50] and based on data collected from one of the largest tertiary care public sector hospital located in India. Being a well-equipped tertiary care center, both in terms of infrastructure/human resource (more than 100 health care personnel involved in cancer care delivery) and catering to more than 5000 cancer patients annually, justifies the appropriateness of unit cost estimates calculated based on the study hospital. [51]

A limitation of the study was the use of certain parameter values derived from a mathematical model. Due to unavailability of any empirically derived estimates on the natural history of progression in undiagnosed cases of cancer as well as their probability of showing symptoms from India, parameter values derived from a mathematical model developed by Myers et al were used. [16] These estimates have also been used to parameterize models to evaluate cervical cancer prevention strategies in Thailand, United Kingdom and Germany. [13–15] Moreover, since the natural progression of disease is not expected to vary by region, these estimates were considered appropriate. Similarly, due to lack of Indian specific data on incidence of HPV infection, age specific HPV incidence rates were derived based on data of HPV infection in a vaccinated cohort of adolescent girls. [28] Both these derived estimates could have affected the valuation of health outcomes. However, it was seen that our model predicted life time risk of incurring cervical cancer of 2.09% for no screening scenario, which was almost similar to the lifetime risk of cervical cancer reported in data from Indian cancer registries. [21] Further, these derived estimates were varied in sensitivity analysis and thus our study findings are robust.

Another limitation of the current analysis is that due to non- availability of the incidence data for HPV infection, we had to omit considering cervical cancers caused by types other than HPV 16/18. We acknowledge that screening strategies are likely to detect even more cases (if cancer cases caused by non 16/18 HPV variants are also considered) than what has been shown in the current analysis. Hence, the magnitude of disease reduction of cervical cancer screening would be more than what has been shown in our analysis, as the current analysis focuses only on HPV 16/18. In order to predict this accurately, the data on incidence of precancerous lesions in India is required. However, the national cancer registry program generates estimates on the incidence of cancer cases in India. [21] There is no systematic recording of incidence of precancerous lesions. Similarly, while the data on incidence of HPV 16 & 18 infection is available, the incidence estimates for other HPV infection is not present. As a result, it was not possible to accurately model the cervical cancer cases caused by the reasons other than the high- risk HPV infection. Nevertheless, we would like to mention that the two HPV types considered in our analysis account for almost 85% of the total cervical cancer cases in India, making findings robust enough.

In case of the policy of vaccination and screening combined, the additional benefit of screening besides vaccination are accrued because of three reasons, firstly, vaccinated women would benefit from getting non- HPV 16/18 cancers detected, secondly, unvaccinated women would benefit from getting all cervical cancers detected, and thirdly, vaccinated women on which vaccine proved inefficacious (7%) would benefit from all cancer getting detected. Although our model accounts for 85% of the benefits due to the second and the third reason mentioned above, it doesn’t capture the benefits resulting from a vaccinated woman having non- HPV 16/18 cancers detected. As a result, for the scenario of vaccination and screening combined, the prevention offered particularly by the screening has slightly been undervalued. Our model has underestimated about 8% of the cervical cancer cases that the policy of vaccination and screening combined would actually be preventing (S4 File). Although this underestimation is likely to improve the ICER value for the policy of vaccination and screening combined, yet the exact effect is not straightforward, as factors like additional cost of diagnostic tests (colposcopy, biopsy etc.), averted cost of cancer treatment, and QALYs contributed by these prevented cases will play an important role in determining the ICER. In such as case, the conclusion of the present study results will be further enhanced. However, since several of these non-high risk precancerous lesions may not progress of develop cancer, it is likely to result in an increase in false-positives, which will increase the cost of screening and hence increase the ICER value. In order to test the possibility of increase in ICER with increase in false positives, we undertook univariate sensitivity analysis for specificity. We found that screening with VIA every 5 years remains cost effective even till the specificity is reduced to 15% (S1 File of S1 Fig). Hence, we believe that while the analysis would be more robust, if non high risk HPV variants are also included in the study, however, the results of present analysis in terms of their conclusion about VIA 5 years being most cost effective is valid. Lastly, since we have not used a dynamic transmission model, we have not accounted for herd immunity effects of the vaccination.

### Conclusion

Among various screening strategies, screening with both VIA 5 yearly and VIA 10 yearly is cost effective at 1-time per capita GDP, with VIA every 5 years providing greater health benefits as compared to VIA 10 years. Hence, as of now for NPCDCS, we recommend VIA 5 years as the strategy for screening cervical cancer in India. The evidence from our analysis suggests that vaccination is also very cost- effective for prevention of cervical cancer in India. In the long run, a comprehensive strategy of immunizing adolescent girls for HPV along with their screening with VIA between 30 and 65 years of age appears to be a cost- effective strategy at both 5 yearly and 10 yearly frequency. However, it is not a decision that needs to be made until about 15 years from now when the first cohorts of vaccinated women will reach age 30. By that time, we will know about what vaccine coverage level was achieved, and we will also know a lot more about India’s HPV epidemiology, how screening technologies may have advanced, how prices have changed, and how the cost-effectiveness threshold has evolved. Therefore, a reassessment is recommended after 15 years from now, before a decision of this regard is made.

## Supporting information

S1 File (DOCX)

S2 File (DOCX)

S3 File (DOCX)

S4 File (DOCX)
