# Supplementary material for: Cost effectiveness of strategies for cervical cancer prevention in India
Source: PLoS One. 2020 Sep 1;15(9):e0238291. doi: 10.1371/journal.pone.0238291 (PMC7462298; doi:10.1371/journal.pone.0238291)
Supplement: S1 File — (DOCX) [file pone.0238291.s001.docx]

**S1 Supporting information**

**S1 Fig: Change in ICER value (of screening strategy with visual inspection with acetic acid (VIA) every 5 years) with variation in the sensitivity and specificity of VIA**

**
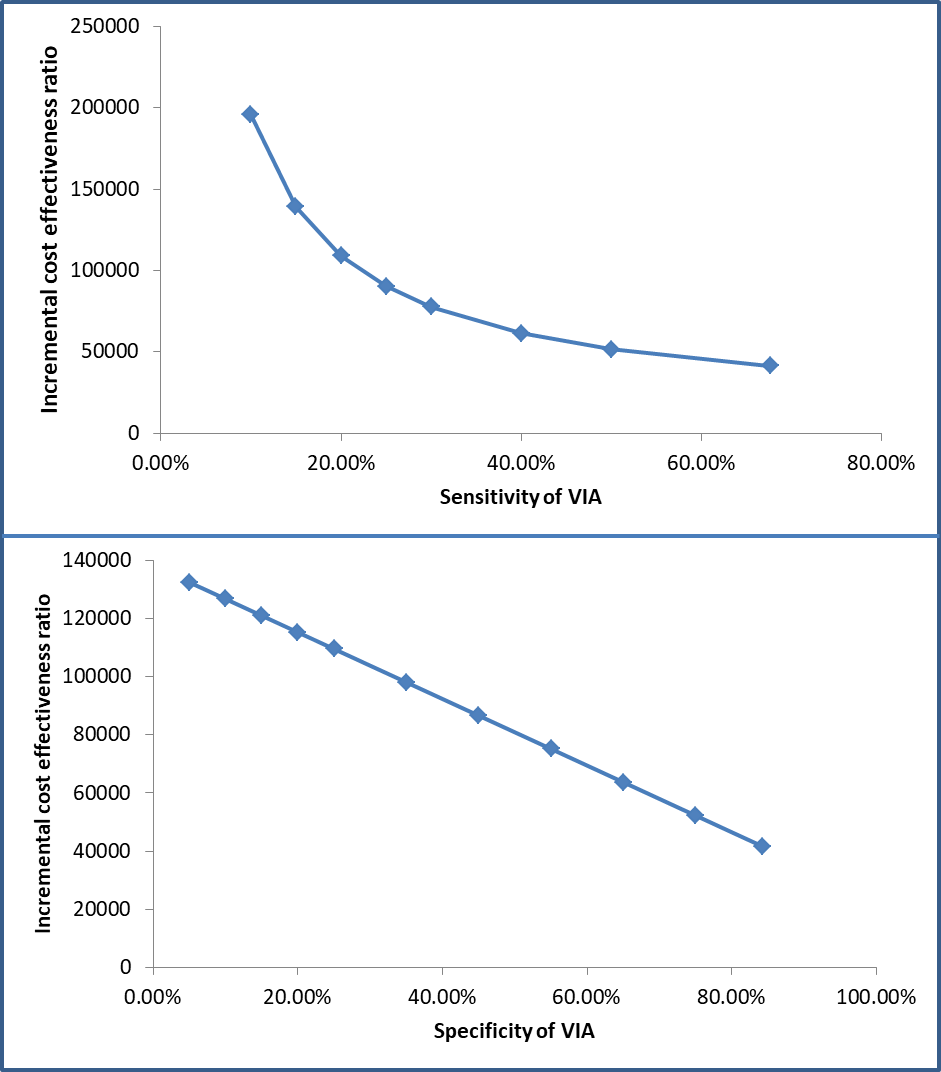
**

**S2 Fig: Cancer cases and death averted in a cohort of 1 lakh women (among income groups) screened with visual inspection with acetic acid every 5 years**

**S1 Table: Model parameters**

| Parameter | | Value |
| --- | --- | --- |
| Age specific all-cause mortality in India | 30-34 years | 0.00149 |
|  | 35-39 years | 0.00198 |
|  | 40-44 years | 0.00282 |
|  | 45-49 years | 0.00379 |
|  | 50-54 years | 0.00761 |
|  | 55-59 years | 0.01025 |
|  | 60-64 years | 0.01631 |
|  | 65-69 years | 0.02513 |
|  | 70-74 years | 0.03978 |
|  | 75+ years | 0.20775 |
| Coverage rates | Opportunistic screening | 0.01 |
|  | Screening with VIA | 0.8 |
|  | Screening with Pap smear | 0.8 |
|  | Screening with HPV DNA | 0.8 |
|  | Colposcopy following screening | 0.9 |
|  | Treatment for precancerous lesions following colposcopy and biopsy | 0.9 |
|  | Treatment for invasive cancer following colposcopy and biopsy | 0.9 |
| Treatment pattern for precancerous lesions | Proportion of women with CIN 1 lesion treated with cryotherapy | 0.6875 |
|  | Proportion of women with CIN 1 lesion treated with LEEP | 0.3125 |
|  | Proportion of women with CIN 2 lesion treated with cryotherapy | 0.2427 |
|  | Proportion of women with CIN 2 lesion treated with LEEP | 0.5317 |
|  | Proportion of women with CIN 2 lesion treated with surgery | 0.2254 |
| Treatment pattern for invasive cancer | Proportion of stage I patients getting surgical treatment only | 0.33 |
|  | Proportion of stage I patients getting radiotherapy followed by brachytherapy and chemotherapy | 0.33 |
|  | Proportion of stage I patients getting radiotherapy and brachytherapy preceded by surgery | 0.34 |
|  | Proportion of stage II patients getting radiotherapy followed by brachytherapy and chemotherapy | 0.67 |
|  | Proportion of stage II patients getting radiotherapy and brachytherapy preceded by surgery | 0.33 |
|  | Proportion of stage III patients getting radiotherapy followed by brachytherapy and chemotherapy | 1 |
|  | Proportion of stage IV patients getting radiotherapy only | 0.25 |
|  | Proportion of stage IV patients getting radiotherapy followed by brachytherapy and chemotherapy | 0.5 |
|  | Proportion of stage IV patients getting radiotherapy followed by Chemotherapy | 0.25 |
| Stage specific recurrence rates following treatment for cervical cancer | Stage 1 | 0.1 |
|  | Stage 2 | 0.25 |
|  | Stage 3 | 0.42 |
|  | Stage 4 | 0.76 |
| Treatment pattern for recurrence | Proportion of patient with recurrence treated with radiotherapy | 0.3 |
|  | Proportion of patient with recurrence treated with chemotherapy | 0.3 |
|  | Proportion of patient with recurrence treated with basic support only | 0.4 |

**S2 Table: Comparison of HPV incidence rates as derived in the present model with that of the mathematical model by Myers et al.**

| Age groups | HPV infection incidence rate (per person) derived in the present model | HPV infection incidence rate (per person) by Myers et al |
| --- | --- | --- |
| 30-34 years | 0.06 | 0.01 |
| 35-39 years | 0.047 | 0.01 |
| 40-44 years | 0.047 | 0.01 |
| 44-49 years | 0.046 | 0.01 |
| 50-54 years | 0.0125 | 0.005 |
| 55-59 years | 0.0125 | 0.005 |
| 60-64 years | 0.0125 | 0.005 |

**S3 Table: Per capita reduction in out of pocket expenditure with implementation of various screening strategies**

| Screening strategy | | Life time per capita reduction in out of pocket expenditure | |
| --- | --- | --- | --- |
|  |  | INR | USD |
| Visual inspection with acetic acid | **3 Years** | 641.66 (284-1447) | 9.69 (4.39-21.90) |
|  | **5 Years** | 587.83 (245-1374) | 8.88 (3.70-20.8) |
|  | **10 Years** | 530.68 (212-1307) | 8.02 (3.20-19.74) |
| PAP smear | **3 Years** | 632.72 (274-1397) | 9.56 (4.15-21.90) |
|  | **5 Years** | 578.37 (241-1323) | 8.74 (3.65-19.87) |
|  | **10 Years** | 527.38 (204-1266) | 7.97 (3.09-19.12) |
| HPV DNA test | **3 Years** | 659.46 (310-1549) | 9.96 (4.68-23.40) |
|  | **5 Years** | 600.76 (271-1425) | 9.07 (4.09-21.52) |
|  | **10 Years** | 539.82 (227-1330) | 8.15 (3.43-20.09) |

*Values in parenthesis represent 2.5^th^ and 97.5^th^ percentile; US$: Unites States Dollar; INR: Indian rupees

**S4 Table: Total cost incurred with implementation of various screening strategies**

| **Screening strategy** | | **Health system cost in INR million (%)** | **OOP expenditure in INR million (%)** | **Total** |
| --- | --- | --- | --- | --- |
| **No organized screening** | | 81 (49.8) | 82 (50.2) | 163 |
| **Visual inspection with acetic acid*** | **3 Years** | 771 (97.7) | 18 (2.3) | 789 |
|  | **5 Years** | 439 (95) | 23 (5) | 462 |
|  | **10 Years** | 291 (91) | 28 (9) | 319 |
| **PAP smear*** | **3 Years** | 835 (97.7) | 19 (2.3) | 855 |
|  | **5 Years** | 483 (95) | 24 (5) | 508 |
|  | **10 Years** | 316 (91.5) | 29 (8.5) | 345 |
| **HPV DNA test*** | **3 Years** | 1086 (98.6) | 15 (1.4) | 1101 |
|  | **5 Years** | 626 (97) | 21 (3) | 647 |
|  | **10 Years** | 396 (93.6) | 27 (6.4) | 423 |
| **HPV vaccination alone^@^** | | 121 (85) | 22 (15) | 143 |
| **HPV vaccination along with screening (VIA every 5 years)^@^** | | 337 (98) | 6 (2) | 343 |
| **HPV vaccination along with screening (VIA every 10 years)^@^** | | 252 (97) | 8 (3) | 260 |

*Future costs were discounting from 30 year onwards; ^@^ Future costs were discounted from 11 years onwards; INR: Indian rupees

**S5 Table: Cost summary with introduction of vaccination alone and in combination with screening**

| **Strategies** | **Cost in INR million** | | | |
| --- | --- | --- | --- | --- |
|  | **Screening cost** | **Vaccination cost** | **Treatment cost** | **Total** |
| **No vaccination** | 17 (11-23) | - | 92 (48-171) | 109 (64-191) |
| **HPV Vaccination** | 14 (9-20.5) | 92 | 36 (19-68) | 143 (103-195) |
| **HPV vaccination along with screening (VIA 5 yearly)** | 229 (164-319) | 92 | 21 (11-38) | 343 (268-439) |
| **HPV vaccination along with screening (VIA 10 yearly)** | 141 (102-192) | 92 | 26 (14-48) | 260 (206-327) |

*Values in parenthesis represent 2.5^th^ and 97.5^th^ percentile; INR: Indian rupees; Future costs were discounted from 11 years onwards

**S6 Table: Cost of cervical cancer screening in India as reported in various studies**

| Parameter | Present study | Diaz et al (2008), Goldie et al (2005) | | Legood et al (2005) | |
| --- | --- | --- | --- | --- | --- |
|  | **INR (2017)** | **I$ 2005** | **Converted to INR 2017** | **US$ 2005** | **Converted to INR 2017** |
| Cost per woman screened with VIA test | 344 | 1.25 | 32.21 | 3.917 | 396.94 |
| Cost per woman screened with Cytology test | 652 | 3.69 | 96.11 | 6.609 | 773.88 |
| Cost per woman screened with HPV DNA test | 980 | 10.30 | 265.73 | 11.779 | 1404.49 |

*I$: International Dollar; US$: United States Dollar; INR: Indian Rupees; VIA: Visual inspection with acetic acid.
