# Supplementary material for: Cost effectiveness of strategies for cervical cancer prevention in India
Source: PLoS One. 2020 Sep 1;15(9):e0238291. doi: 10.1371/journal.pone.0238291 (PMC7462298; doi:10.1371/journal.pone.0238291)
Supplement: S2 File — (DOCX) [file pone.0238291.s002.docx]

**S2 Supporting information: Cost of Camp-Based Screening for Cervical, Breast and Oral cancer**

This draft presents the methods and results of the study undertaken to assess the cost of implementing camp based screening for cervix, breast and oral cancer in the Villupuram district of Tamil Nadu. As part of the present screening program program, all the eligible women in the age group of 30-65 years were screened for cancer cervix with VIA as well as with HPV DNA test. Samples were also taken for Pap smear for those women who were screened positive with HPV DNA. Screening was organized for 2-3 days at each of the selected village, preceded by 1-2 day awareness activity and enumeration of the eligible women in the respective villageby the social workers,. Sample collection/visual inspection was done by a trained health worker (equivalent to ANM). HPV DNA samples were processed at the district level itself by the trained laboratory technicians. However, Pap smears were processed at the cytopathology laboratory of a tertiary care hospital located in Chennai.

Costs were assessed following the economic costing approach and bottom-up methodology. All costs pertained to the financial year 2016-2017. Data on annual consumption of both capital and recurrent resources, spent on the provisioning of camp based screening during the reference year of 2016-17 were collected and analyzed. The total annual cost of this screening activity along with its distribution in terms of inputs and type of services has been computed. In addition, unit costs of specific services have also been estimated.

A total of 10,578 women underwent camp based screening, of which 9,173 women were screened for cervical cancer with VIA/VILI as well as HPV DNA test, as shown in table 1. Of the total women screened for cervical cancer, 5,260 women were also screened with Pap test. A total annual of INR 17,372,512 (INR 17.3 million of 1.73 crores) was spent in organising the screening, including the cost on laboratory processing for HPV DNA and Pap test. Input wise distribution of this annual cost has been shown in table 2 and figure 1. It was seen that of the overall cost, around 43% (INR 7,530,941) was spent on the salaries of the human resource, followed by spending on the purchase of consumables (41%; INR 7,091,592) and equipment (7%; INR 1,201,388).

In terms of distribution of total cost in terms of specific services, more than half (55%) of the overall cost was spent on sample collection (9.6%, INR 1,675,903) and laboratory processing of the HPV DNA (37%; INR 6,397,502) and Pap test (8.9%; INR 1,541,301) (Table 3 and figure 2). This was followed by expenses for carrying out the household survey (15%; INR 2,624,619) and screening for breast cancer (9.8%; INR 1,702,191). Further, a total of INR 856,496 (5%) and INR 585,449 (3.4%) were spent on transport and administrative activities respectively.

With respect to cost per patient screened, INR 161 and INR 22 was spent for screening a women for breast and oral cancer (table 3). Specifically, unit cost (per patient cost) of screening a women with either of the 3 screening strategies for cervical cancer has been shown in table 4. Unit cost of screening a patient with VIA/VILI was INR 344 of which INR 103 was spent on the visual inspection and rest (INR 241) on the support activities. Further, unit cost of INR 980 was spent on screening a women with HPV DNA, of which INR 162 and INR 578 was spent on sample collection and laboratory processing. Similarly, INR 652 was consumed per women screened with Pap test, of which INR 118, INR 293 and INR 241 was spent on sample collection, lab processing and support activities respectively. Support activities include organising camp, administration, registration, transport, supervision and miscellaneous activities.

Input wise distribution of the total cost spent on the laboratory processing of the HPV DNA and Pap test has been shown in table 5 and 6 respectively. In case of HPV DNA, major spending of 93% (INR 5,975,476) was on the purchase of consumables. While in the case of lab processing for Pap test, major portion of 72% (INR 1,110,941) was spent on the salaries of the pathologist and lab technicians.

**S1 Table: Number of patients screened during the reference period from April 2016 to March 2017**

| **Variable** | **N** |
| --- | --- |
| Number of patients screened | 10,578 |
| Patients screened with VIA/VILI as well as HPV DNA | 9,173 |
| Patient screened with PAP test | 5,260 |
| VIA/VILI positive patients | 3,890 |
| HPV DNA positive patients | 544 |
| Pap positive patients | 159 |

**S2 Table: Input wise distribution of total annual cost of camp based screening for cervix, breast and oral cancer during the financial year of 2016-17**

| Inputs | Annual cost (INR) |
| --- | --- |
| Human resource | 7,530,941 |
| Space/Building | 538,775 |
| Furniture | 45,345 |
| Equipment | 1,201,388 |
| Consumables | 7,091,592 |
| IEC Material | 68,619 |
| Stationary | 86,056 |
| Overheads | 809,796 |
| Total cost | **17,372,512** |

**S1 Figure: Input wise distribution of total annual cost of camp based screening for cervix, breast and oral cancer**

**S3 Table: Total annual and unit cost of specific services of camp based screening for cervix, breast and oral cancer**

| Specific activities | Annual cost (INR) | Unit cost (cost per patient) in INR |
| --- | --- | --- |
| HPV DNA laboratory processing | 6,397,502 | 578 |
| Survey/IEC | 2,624,619 | 248 |
| Screening of Breast Cancer | 1,702,191 | 161 |
| Screening of Cervical Cancer (sample collection) | 1,675,903 | 183 |
| Pap smear (laboratory processing) | 1,541,301 | 293 |
| Transport | 856,496 | 81 |
| Administration | 585,449 | 55 |
| Research/Report writing | 452,045 | 43 |
| Organising for the camp | 348,000 | 33 |
| Registration of patients | 345,322 | 33 |
| Supervision | 301,364 | 28 |
| Screening of Oral Cancer | 231,834 | 22 |
| Meetings | 201,777 | 19 |
| Miscellaneous | 108,708 | 10 |
| Total | **17,372,512** | **1642** |

**S2 Figure: Services wise distribution of total annual cost of camp based screening for cervix, breast and oral cancer**

**S4 Table: Unit costs of various screening strategies for cervical cancer for camp based screening**

| Screening strategy | Per patient cost | | | |
| --- | --- | --- | --- | --- |
|  | **Sample collection/visual inspection** | **Laboratory processing** | **Support**  **activities*** | **Total** |
| VIA/VILI | 103 | NA | 241 | 344 |
| Pap test | 118 | 293 | 241 | 652 |
| HPV DNA | 162 | **578** | 241 | 980 |

*Support activities include organising for the camp, administration, registration, transport, supervision and miscellaneous activities.

**S5 Table: Input wise distribution of total annual cost incurred on laboratory processing of HPV DNA test for cervical cancer screening**

| Inputs | Annual cost in INR (%) |
| --- | --- |
| Human resource | 132,000 (2) |
| Capital | 15,654 (0.2) |
| Furniture | 12,372 (0.2) |
| Equipment | 177,409 (2.8) |
| Consumables | 5,975,476 (93.4) |
| Overheads | 84,592 (1.3) |
| Total cost | **6,397,502** |

**S6 Table: Input wise distribution of total annual cost incurred on laboratory processing of Pap smear for cervical cancer screening**

| Inputs | Annual cost in INR (%) |
| --- | --- |
| Human resource | 1,110,941 (72) |
| Capital | 315,509 (20.5) |
| Furniture | 8,141 (0.5) |
| Equipment | 15,489 (1) |
| Consumables | 91,221 (5.9) |
| Total cost | **1,541,301** |
