# Supplementary material for: Cost effectiveness of strategies for cervical cancer prevention in India
Source: PLoS One. 2020 Sep 1;15(9):e0238291. doi: 10.1371/journal.pone.0238291 (PMC7462298; doi:10.1371/journal.pone.0238291)
Supplement: S3 File — (DOCX) [file pone.0238291.s003.docx]

**S3 Supporting information:** **Methodology note on cost of treatment for cervical cancer in India**

**Study setting**

The present study was conducted in the Departments of obstetrics & gynaecology (OBG) and radiation oncology of a tertiary care public sector hospital located in North India. The hospital has facilities for provision of surgical care, radiotherapy, brachytherapy and chemotherapy for cancer treatment. The department of OBG has a total of 16 gynaecologists and 70 resident doctors involved in providing specialised health care. Similarly, the radiation oncology department has 10 oncologists, 23 resident doctors, 6 medical physicists and 27 technical staff members involved in the delivery of radiation therapy for cancer treatment. Further, among these staff members, 1 radiation oncologist and 5 resident doctors are specifically involved in providing treatment to gynaecological cancers. Eight radiotherapy machines i.e., 2 using Cobalt-60, 4 using linear accelerators and 2 brachytherapy machines were used for providing radiotherapy at the time of data collection.

**Flow of treatment process**

Patients with symptoms of suspected cervical cancer first reports to the outpatient clinic (OPD) of the OBG department. After clinical investigations (like biopsy, blood tests, etc.), the modality of treatment to be given to the patient is decided at this level. Surgical treatment is offered in the OBG department itself. For further management i.e. radiotherapy, brachytherapy and chemotherapy patients are referred to the department of radiation oncology.

**Data collection**

**Health system cost**

Health system cost i.e. cost incurred to the hospital, was assessed following the concept of economic costing and bottom-up approach. (1, 2) Using this methodology, the first step involved identification and classification of cost centres in terms of those directly involved in cancer treatment. These included out-patient clinic, operation theatre, in-patient ward and radiotherapy units). Similarly, the supportive or indirect cost centers (Laboratory, radio-diagnosis units, pharmacy, dietetics, laundry, etc. (1) After identification of respective cost centers, data on the quantity of various inputs i.e., both capital and recurrent resources spent on the delivery of services was collected for the reference year of 2016-17.

Facility maps obtained from the engineering department of the hospital were reviewed for assessing the dimensions of space and building. Further, the non-consumable stock registers were reviewed for assessing the quantity of various medical/non-medical equipment and furniture items available in the department. Similarly, recurrent resources in the form of drugs, consumables, surgical supplies, sanitary and stationary items were estimated by reviewing the consumable stock registers, indents, vouchers and pharmacy records. Data on the salaries (inclusive of all the annual incentives) received by each of the staff members, both partly or completely involved in the cancer treatment, was assessed from the payslips available from the accounts department. Patient files were assessed for details on the number of various diagnostic tests prescribed to the patient of cervical cancer. Following identification and measurement of quantity of inputs, data on the service output produced by each of the cost centers (in the form of the number of out-patient consultations, in-patient admissions, surgeries, radiotherapy sessions, etc.) was assessed from the reports annual and records of the department.

The next step involved assigning a monetary value to each of the inputs. For estimating space costs, the current market rental price of a similar space was assessed based on key informant interviews. The procurement prices of equipment, drugs and consumables, as obtained from the procurement department and central store of the study hospital was used. Procurement price was not available for furniture items, for which market prices were used. The cost of overheads like water, maintenance, laundry and dietetics was obtained at the overall hospital level. Further, data on actual consumption of expenditure on electricity in kilowatt-hour in each of the rooms of the department was obtained based on the assessment by the electrical engineers of the study hospitals. The cost of various diagnostic tests, as reported in a recent study conducted in the same hospital was used. (3)

Both the medical and the technical staff members involved in cervical cancer treatment were interviewed for assessing their time spent on various activities. Specifically, the medical staff was interviewed for time spent on activities carried out both on regular basis (outpatient consultation, inpatient care, operation theatre, radiotherapy treatment, etc.) as well as on fixed interval (meetings, teaching/training, etc.) i.e., weekly, monthly, annually, etc. Similarly, technical staff related to radiotherapy treatment were interviewed for their time spent on planning (like CT simulation, contouring, dosimetry, etc.), quality assurance and radiotherapy delivery. Alongside these interviews, observational data was collected for per patient time spent on CT simulation, planning (including contouring and dosimetry) and radiotherapy delivery. A total 3 faculty members, 4 senior and 4 junior residents, a medical physicist and 3 technical staff members were interviewed and subsequently observed.

**Out of Pocket expenditure**

“Cost of Illness” approach was followed, which classifies OOP expenditure into direct (including both direct health care and direct non-health care expenditure) and indirect health-care expenditure. (4) Direct health expenditure included expenses incurred on user fee for procedure, diagnostic tests or other services, drugs, consumables etc. Further, the expenditure on transportation, boarding/lodging and food, were considered under direct non-health expenditure. As the main aim of the study was to estimate the cost of cancer treatment to inform price-setting, the indirect health care expenditure incurred by the households was not estimated.

Data on OOP expenditure was elicited from 2 groups of patients (n= 248). The first group comprised of patients (n= 64) who were recruited at the time of registration in the department of radiation oncology and were prospectively followed up till the entire duration of their treatment. The second group consisted of those patients (n= 184) who had completed their treatment (within the last 6 months) and were retrospectively interviewed at the time of their follow-up visit. Patients from both the groups were first of all contacted in the outpatient clinic (OPD) of the Radiotherapy Department. For the first set of patients, all new registrations of cervical cancer, during the period of data collection, were approached on a continuous daily basis for recruitment in the present study. For the second set, all those post-operative cancer cases, visiting the OPD clinic for their follow up visits were asked for willingness to participate. A consecutive sampling was followed till the number of patients to be included in the study was recruited.

The recruited patients were interviewed based on a pre-tested semi-structured interview schedule, adapted from previous studies done in the similar settings. (4, 5, 6) It included information on socio-demographic characteristics, duration of treatment, consumption expenditure, insurance status, OOP expenditure incurred on diagnosis/treatment and coping mechanisms for dealing with the same. Payment receipts and bills were checked where available from the participants to validate the reported expenditure. Expenditure incurred on pre-radiotherapy treatment (in the gynaecology department) and specifically on surgery (if any), was elicited retrospectively from both the groups. If the patient had taken any treatment before coming to the study hospital, OOP expenditure on account of the same was also recorded.

**References:**

1. Drummond ME, Stoddard GL, Torrance GW. Methods for the Economic Evaluation of Health Care Programmes.
2. Chapko MK, Liu CF, Perkins M, Li YF, Fortney JC, et al. (2009) Equivalence of two healthcare costing methods: bottom-up and top-down. Health Econ 18: 1188–1201. pmid:19097041.
3. Sangwan A, Prinja S, Aggarwal S, Jagnoor J, Bahuguna P, Ivers R. Cost of Trauma Care in Secondary- and Tertiary-Care Public Sector Hospitals in North India. Applied health economics and health policy. 2017.
4. Chauhan AS, Prinja S, Ghoshal S, Verma R, Oinam AS. Cost of treatment for head and neck cancer in India. Plos One [Internet]. 2018Nov [cited 20Jul2018];13(1).
5. Prinja S, Jagnoor J, Chauhan AS, et al (2016). Economic Burden of Hospitalization Due to Injuries in North India: A Cohort Study. International journal of environmental research and public health, 13, pii: E67.
6. Prinja S, Bahuguna P, Duseja A, et al (2017a). Cost of Intensive Care Treatment for Liver Disorders at Tertiary Care Level in India. PharmacoEconomics - Open.
