# Supplementary material for: Cost effectiveness of strategies for cervical cancer prevention in India
Source: PLoS One. 2020 Sep 1;15(9):e0238291. doi: 10.1371/journal.pone.0238291 (PMC7462298; doi:10.1371/journal.pone.0238291)
Supplement: S4 File — (DOCX) [file pone.0238291.s004.docx]

**S4 Supporting information: Methodological note on the calculation of the 8% underestimate of cases for the scenario of vaccination+ screening**

Assuming that a cohort of women without vaccination or screening will develop 100 cervical cancer cases (all types) over its lifetime. Based on the evidence that 85% of India's cervical cancers are due to 16/18, 85 of these cases will occur by the infection from HPV 16/18, and remaining 15 cases will be due the non-16/18 high-risk types. Considering the vaccine coverage and efficacy at 70% and 93%, respectively, about 56 of the 85 cases due to 16/18 would be prevented with vaccination. And 29 cases due to 16/18 and 15 cases due to non-16/18 high risk types will remain, which will then be benefitted or prevented by screening. In the present analysis, screening with VIA 5 years (the intervention which is found the most- cost effective among all screening alternatives) prevents 41% of the cases. So, screening would further detect about 12 cases of HPV 16/18 (29*0.41) and 6 cases of non-16/18 high risk types (15*0.41), i.e., a total of 18 cases. So total cancers cases averted due to vaccination+ screening would be 56+18= 74. However, our model captures only 56 and 12 cases (HPV 16/18 only) that is equal to 68 cases. Therefore, for the scenario of vaccination+ screening, our model underestimates about (74-68/74) 8% of the cervical cancer cases that the policy would actually be preventing.
